# A structured list of laboratory tests for screening the possible causes of small fiber neuropathy in clinical practice

**DOI:** 10.3389/fneur.2025.1679316

**Published:** 2026-01-12

**Authors:** Jean-Pascal Lefaucheur, Thierry Gendre, Damien Sène

**Affiliations:** 1 Department of Clinical Neurophysiology, Henri Mondor University Hospital, AP-HP, Créteil, France; 2 UR 4391, ENT Team, Faculty of Health, Paris Est Creteil University, Créteil, France; 3 Department of Neurology, Henri Mondor University Hospital, AP-HP, Créteil, France; 4 Department of Internal Medicine, Lariboisière University Hospital, AP-HP, Paris, France

**Keywords:** blood test, diagnosis, dysautonomia, investigation, neuropathic pain, peripheral neuropathy, small-diameter nerve fibers

## Abstract

Small fiber neuropathies (SFN) are increasingly recognized as the cause of various sensory and autonomic disorders. Different tests exist to enable the objective diagnosis of SFN, but these tests generally do not identify a possible etiology. However, finding the cause of SFN is the best way to implement effective treatment. Thus, the etiological assessment must be as exhaustive as possible so as not to miss a curable cause of SFN. This search is based primarily on patient’s history and clinical examination but may also require additional laboratory investigations. The objective of this article is to provide recommendations to help practitioners rationalize these investigations, mainly blood tests, with the aim of identifying the possible cause of SFN in a given patient. The first-line blood tests we generally recommend help identify two main categories of possible etiologies of SFN: firstly, metabolic and endocrine causes (diabetes, prediabetes, metabolic syndrome, insulin resistance, vitamin B disorders, renal or hepatic insufficiency, and thyroid diseases), and secondly, immunological, inflammatory, and infectious causes (autoimmune connective tissue diseases, celiac disease, monoclonal gammopathy, sarcoidosis, and viral infections). As a second-line approach, we propose complementary investigations that should be considered in more specific clinical situations. An algorithm is presented, summarizing the sequence of investigations to be performed to guide clinicians in their diagnostic approach to SFN.

## Introduction

Small fiber neuropathy (SFN) is a peripheral nervous system disorder based on specific lesions of small-diameter nerve fibers that mediate thermal or pain sensations and distal autonomic nervous regulation. These neuropathies are increasingly recognized and are clinically characterized by alterations in thermal and pain sensitivity, a possible association with neuropathic pain symptoms, such as burning or needle-prick sensations or itching, and autonomic signs (1, 2). A number of complementary histological or neurophysiological investigations can be used to objectify small nerve fiber lesions (loss of quantity or function) and confirm the diagnosis of SFN (3, 4). However, this diagnosis consists only of a part of the management of affected patients. The most important is the etiological diagnosis of SFN, which remains a challenge in clinical practice (5, 6). In the case of large fiber neuropathies, the etiological diagnosis can be established or at least suspected on the complementary examinations that are used for the diagnosis of the neuropathy (pattern of neurophysiological abnormalities in nerve conduction study or neuropathological abnormalities in nerve biopsy study). Conversely, this is not the case for the examinations used for the diagnosis of SFN (except for the demonstration of amyloid deposits in skin biopsy (7)). It is therefore essential to perform a certain number of additional laboratory tests to find the possible causes of SFN in patients.

As working in tertiary centers for the management of SFN, we are regularly asked by our colleagues to know what complementary assessment should be performed for this purpose. Based on our clinical experience and a review of the literature, this article therefore aims to report on our current strategy for researching the possible etiology of SFN, in order to help our colleagues faced with this question.

## Methodology

The search for the possible etiology of SFN relies primarily on patient’s history and clinical examination. For example, a history of medication use can provide evidence of a toxic cause of SFN (e.g., in the context of chemotherapy-induced peripheral neuropathy). A familial history may suggest a genetic cause. Furthermore, associated clinical signs or symptoms may indicate a picture clearly in favor of a specific disease (diabetes, metabolic syndrome, Fabry disease, amyloidosis, immune-mediated systemic disorders, etc.). Therefore, this review aims only to present a comprehensive battery of ancillary tests, which can be performed to search for a possible cause of SFN, even in the absence of suggestive clinical information. The tests to be considered in such an etiological search are essentially blood tests.

In order to formulate a relevant proposal, we initially considered categorizing the tests according to the prevalence of each specific etiology of SFN. Unfortunately, very disparate prevalence values were reported in the only two studies we identified (5, 6) that presented detailed results of large-scale blood tests to determine the possible etiology of SFN (Table 1).

**Table 1:** Prevalence of abnormal blood test results for the etiological diagnosis of small fiber neuropathy in two previously published studies.

Disease condition	Prevalence in Lang et al. (5)	Prevalence in de Greef et al. (6)
Diabetes mellitus	5.5%	7.7%
Prediabetes	25%	9.7%
Hypertriglyceridemia	24.7%	Unexplored
Vitamin B1 deficiency	Unexplored	2.4%
Vitamin B6 toxicity	Unexplored	5.2%
Vitamin B9 deficiency	2%	Unexplored
Vitamin B12 deficiency	1.5%	4.7%
Renal insufficiency	2.5%	0.4%
Hypo/hyperthyroidism	6.2%	Unexplored
Alcohol abuse	Unexplored	3%
Hepatopathy	14.8%	Unexplored
Haemochromatosis	Unexplored	0.3%
Lupus	27.5%	Unexplored
Hypocomplementemia	15.7%	Unexplored
Monoclonal gammopathy	3.9%	1.4%
Sarcoidosis	0%	3%
Sjögren’s disease	9.2%	1.3%
Celiac disease	3.5%	0.5%
Human immunodeficiency virus	Unexplored	0%
Hepatitis C virus	1.1%	Unexplored
Lyme disease	8.7%	Unexplored
Fabry disease	Unexplored	0%
Sodium channel mutation	Unexplored	16.7%

A second approach we initially considered for test categorization was based on clinical characteristics, such as: (i) age of onset; (ii) mode of onset (acute, rapidly progressive, and chronic); (iii) sensory or autonomic predominance; and (iv) distribution of signs and symptoms according to length-dependent or non-length-dependent pattern. However, it proved difficult to establish specific phenotypes of SFN that would allow us to base a categorization and prioritization algorithm for tests solely on the clinical presentation. Indeed, clinical presentations largely overlap in SFN, and the link with specific etiologies is less clear than for large-fiber axonal or demyelinating neuropathies. For example, although cases of non-length-dependent SFN (possibly ganglionopathy) have been described (8), no specific etiology has been identified (glucose metabolism disorders, Sjögren’s syndrome, monoclonal gammopathy, celiac disease, and hepatitis C virus infection have been reported in this series (8)). Nevertheless, the issue of specific SFN phenotypes and clinical contextualization in the choice of etiological tests to be performed has been addressed throughout the text, where relevant.

In fact, it seemed to us that the most relevant criteria for categorizing tests were the following: (i) their ability to reveal a possible, well-defined, and non-rare cause of SFN; (ii) their justification in either the absence or the presence of specific clinical warning signs; and (iii) their ease of performance and accessibility in routine clinical practice. We therefore distinguish two categories of blood tests:

- (i) a first category of tests that can be routinely performed and correspond to well-defined possible etiologies of SFN that may be observed without specific clinical warning signs (Table 2);
- (ii) a second category of tests that correspond to well-defined possible etiologies of SFN, but which complement first-line screening tests or are non-routine tests carried out in a hospital setting, or are associated with specific clinical warning signs (Table 3).

**Table 2:** List of first-line blood tests to investigate the etiology of small fiber neuropathy.

Blood test	For the diagnosis of
Fasting plasma glucose (FPG)	Diabetes, prediabetes, metabolic syndrome
Glycated hemoglobin A1C (HbA1C)	Diabetes, prediabetes
Triglyceride (TG)	Metabolic syndrome (incl. Liver steatosis)
High-density lipoprotein cholesterol (HDL-C)	Metabolic syndrome (incl. Liver steatosis)
Fasting insulin (FIns)	Metabolic syndrome (incl. Liver steatosis)
Serum uric acid (SUA)	Metabolic syndrome (incl. Liver steatosis)
Pyridoxal 5-phosphate (PLP)	Vitamin B6 intoxication
Total circulating B12 (serum cobalamin)	Vitamin B12 deficiency
Creatinine (Cr)/estimated glomerular filtration rate (eGFR)	Chronic kidney disease
Gamma-glutamyl transferase (GGT)	Liver disease (alcohol, sarcoidosis, HCV infection)
Aspartate aminotransferase (AST)	Liver disease (alcohol, HCV infection)
Alanine aminotransferase (ALT)	Liver disease (HCV infection)
Alkaline phosphatase (ALP)	Liver disease (sarcoidosis)
Ferritin (Ferr)	Hemochromatosis, celiac disease
Transferrin saturation (TSAT)	Hemochromatosis
Thyroid stimulating hormone (TSH)	Hypothyroidism
Complete blood cell count (CBC)	B12 deficiency, alcohol, celiac disease, sarcoidosis
Anti-nuclear antibodies (ANA)	Sjögren’s syndrome, systemic lupus erythematosus
Anti-extractable nuclear antigen antibodies (ENA)	Sjögren’s syndrome, systemic lupus erythematosus
Anti-tissue transglutaminase (anti-tTG) IgA	Celiac disease
Serum protein electrophoresis (SPEP)	Monoclonal gammopathy, sarcoidosis
Serum protein immunofixation electrophoresis (SIFE)	Monoclonal gammopathy (incl. AL amyloidosis)
Serum free light chain assay (SFLCA)	Monoclonal gammopathy (incl. AL amyloidosis)
Serum angiotensin-converting enzyme (ACE)	Sarcoidosis (× 2 upper limit of normal)
Calcemia (Calc)	Sarcoidosis
Hepatitis C virus (HCV) serology	HCV infection
Human immunodeficiency virus (HIV) serology	HIV infection

**Table 3:** List of second-line blood tests to investigate the etiology of small fiber neuropathy.

Blood test	If the following condition
75 g oral glucose tolerance test (OGTT) with 1 h and 2 h plasma glucose (1hPG, 2hPG)	Borderline fasting plasma glucose or hemoglobin A1C
Holotranscobalamin (holoTC)	180 pg./mL ≥ total circulating B12 ≤ 350 pg./mL
Methylmalonic acid (MMA)	180 pg./mL ≥ total circulating B12 ≤ 350 pg./mL
Total homocysteine (tHcy)	180 pg./mL ≥ total circulating B12 ≤ 350 pg./mL
Serum folate (vitamin B9)	180 pg./mL ≥ total circulating B12 ≤ 350 pg./mL
Carbohydrate-deficient-transferrin (CDT)	Suspected and unconfessed alcohol abuse
Phosphatidylethanol (PEth)	Suspected and unconfessed alcohol abuse
Anti-double-stranded (anti-native) DNA (anti-dsDNA)	Clinically suspected autoimmune connective tissue disorder without positivity of first-line tests
C3, C4, and CH50 haemolytic units of the complement	Clinically suspected autoimmune connective tissue disorder without positivity of first-line tests
Cryoglobulinemia (Cryo)	Clinically suspected cryoglobulinemia
C-reactive protein (CRP)	Clinically suspected rheumatoid arthritis
Rheumatoid factor (RF)	Clinically suspected rheumatoid arthritis
Anti-cyclic citrullinated peptide (anti-CCP)	Clinically suspected rheumatoid arthritis
Systemic sclerosis (SSc)-related autoantibodies	Clinically suspected systemic sclerosis
Anti-ribonucleoprotein U1 (anti-U1-RNP)	Mixed connective tissue disease
Antineutrophil cytoplasmic antibody (ANCA)	Clinically suspected vasculitis
Anti contactin-associated protein-like 2 (anti-Caspr2)	Painful or acute-onset small-fiber neuropathy
Anti-leucine-rich glioma-inactivated 1 (anti-LGI1)	Painful or acute-onset small-fiber neuropathy
Anti-fibroblast growth factor receptor 3 (anti-FGFR3)	Painful or acute-onset small-fiber neuropathy
Anti-trisulfated heparin disaccharide (anti-TS-HDS)	Painful or acute-onset small-fiber neuropathy
Anti-plexin D1	Painful or acute-onset small-fiber neuropathy
1,25-dihydroxy vitamin D (1,25(OH)_2_D or calcitriol)	Suspected sarcoidosis (clinically, on chest radiology)
Chitotriosidase (CTO)	Suspected sarcoidosis (clinically, on chest radiology)
Soluble form of the interleukin 2 receptor (sIL-2R)	Suspected sarcoidosis (clinically, on chest radiology)
Transthyretin (*TTR*) gene mutation	Suspected amyloidosis (dysautonomia, cardiomyopathy, rapidly evolving neuropathy)
Serum globotriaosylsphingosine (lyso-Gb3 or -GL3)	Suspected Fabry disease in man (child-onset painful acroparesthesia, cardiomyopathy, proteinuria)
Alpha-galactosidase A (*GLA*) gene mutation	Suspected Fabry disease in woman
Sodium voltage-gated channels Nav1.7–1.8-1.9 (*SCN9A*-*SCN10A*-*SCN11A*) or transient receptor potential ankyrin 1 (*TRPA1*) gene mutation	Child-onset or familial paroxysmal painful acroparesthesia or erythermalgia
Collagen type VI alpha 5 (*COL6A5*) gene mutation	Familial neuropathic itch
Hereditary sensory and autonomic neuropathy (HSAN) mutations	Severe sensory loss, ulcerative mutilations, and autonomic disturbances

In all cases, our choices were justified by quoting relevant articles. This work does not, however, constitute a systematic literature review, but within the context of clinical conditions being well-defined possible etiologies of SFN, we selected references of two types:

- (i) firstly, articles formulating recommendations validating the type of blood tests relevant for establishing the specific diagnosis of these clinical conditions;
- (ii) secondly, key articles presenting the largest patient cohorts and providing evidence of the existence of SFN in these specific clinical conditions.

Thus, this work is based on an analytical framework for prioritizing test selection grounded in expert opinion, but supported by selected data from the literature.

## First-line blood tests

Thus, some blood tests can reveal abnormalities whose causal link with the development of SFN is firmly established, even in the absence of specific clinical features. These tests are commonly performed tests and can be prescribed by primary health care providers.

## Metabolic and endocrine causes of SFN

### Diabetes and prediabetes

Fasting plasma glucose (FPG), glycosylated hemoglobin (HbA1C), and 2 h plasma glucose (2hPG) value during a 75 g oral glucose tolerance test (OGTT) are the three tests on which the diagnosis of diabetes and prediabetes can be established according to international recommendations (9):

- FPG ≥ 7.0 mmol/L (126 mg/dL), or HbA1C ≥ 6.5%, or 2hPG ≥ 11.1 mmol/L (200 mg/dL) define diabetes;
- hyperglycemia (5.6 mmol/L (100 mg/dL) ≤ FPG < 7.0 mmol/L (126 mg/dL), i.e., impaired fasting glucose (IFG)), or elevated HbA1C (5.7% ≤ HbA1c < 6.5%), or altered OGTT (7.8 mmol/L (140 mg/dL) ≤ 2hPG < 11.1 mmol/L (200 mg/dL), i.e., impaired glucose tolerance (IGT)) define prediabetes.

While diabetes is an established cause of SFN (10–13), there is still controversy among studies regarding the existence of a higher prevalence of SFN in the context of prediabetes (12, 14). However, this does not call into question the value of FPG and HbA1C in the evaluation of the possible etiology of SFN.

### Metabolic syndrome and insulin resistance

The diagnosis of metabolic syndrome can be established according to various definitions and related sets of criteria (15), as follows:

- elevated waist circumference (with population- and country-specific definitions);
- elevated blood pressure (systolic ≥130 mmHg or diastolic ≥85 mmHg);
- elevated FPG (≥100 mg/dL (5.6 mmol/L));
- elevated triglycerides (TG) (≥150 mg/dL (1.7 mmol/L));
- reduced high-density lipoprotein cholesterol (HDL-C) (≥40 mg/dL (1.0 mmol/L) in males; 50 mg/dL (1.3 mmol/L) in females).

Drug treatment for elevated blood pressure, FPG, TG, or cholesterol being alternate indicators. As for prediabetes, there is controversy among studies regarding the existence of a causal relationship between metabolic syndrome and the occurrence of SFN (12, 16). However, there is sufficient data in the literature to consider that the search for hypertriglyceridemia, at least, makes sense in the etiological diagnosis of SFN (16).

In addition to elevated FPG or TG, reduced HDL-C is part of the blood test criteria for the diagnosis of metabolic syndrome (15). Although the presence of metabolic syndrome per se is not as established as hypertriglyceridemia as a possible cause of SFN, a more comprehensive investigation seems legitimate. In fact, there are various emerging surrogate biological markers of metabolic syndrome, notably reflecting a process of insulin resistance (IR) (17), such as:

- the triglyceride glucose index (TyG) = log ([TG × FPG]/2);
- the atherogenic index of plasma (AIP) = log (TG/HDL-C);
- the metabolic score for insulin resistance (METS-IR) = log ([2 × FPG] + TG) × BMI/log (HDL-C);
- the homeostasis model assessment for insulin resistance (HOMA-IR) = (fasting insulin x FPG)/22.5 (mmol/L) or 405 (mg/dL);
- the quantitative insulin sensitivity check index (QUICKI) = 1/(log(fasting insulin) + log(FPG)).

These different indices, requiring only measurements of TG, FPG, HDL-C and fasting insulin, can help predict the risk and prognosis of cardiovascular complications (18). Since peripheral nerve fiber damage can result from microvascular alterations, the involvement of IR, beyond metabolic syndrome, can be strongly suspected in the development of neuropathies (19), including autonomic SFN (20). Thus, the simple addition of HDL-C and fasting insulin to FPG and TG can provide valuable information to highlight a risk factor for SFN related to metabolic syndrome or IR.

Another biological marker of metabolic syndrome and IR is serum uric acid (SUA) (21). Indeed, hyperuricemia is a good predictor of IR-related complications and has been significantly associated with the risk of developing neuropathy, at least in diabetic patients (22), although it may not be a causal factor (23, 24).

### Vitamin B disorders

Vitamin B disorders are well known causative factors of peripheral neuropathy (25). Mainly vitamin B6 intoxication (26) and vitamin B12 deficiency (27) have been associated with SFN and correspond to our personal experience. Regarding vitamin B6, the serum or plasma pyridoxal 5-phosphate (PLP) level is sufficient for diagnosis, but regarding vitamin B12, the serum cobalamin level (total circulating B12) may not be a reliable marker of neural tissue B12 status (28, 29). As a first-line test, a serum cobalamin value < 180 pg./mL (133 pmol/L) can be considered a marker of vitamin B12 deficiency, while a value > 350 pg./mL (258 pmol/L) can be considered normal (29). If the result is between these two values, it will be necessary to add second-line tests to make the diagnosis (see next chapter). An elevated mean corpuscular erythrocyte volume (MCV) may also be a surrogate marker of vitamin B12 deficiency, making useful to assess complete blood count (CBC).

### Renal or liver disease

Chronic kidney disease (renal insufficiency) is a well-known cause of neuropathy, including SFN (30). The diagnosis is routinely based on serum creatinine level and estimated glomerular filtration rate (eGFR) (31).

SFN can be linked to chronic liver disease and dysfunction through a wide variety of mechanisms. For example, in the context of diabetes or metabolic syndrome, liver dysfunction may correspond to the metabolic dysfunction–associated steatotic liver disease (MASLD) (32). Beyond liver ultrasonography or biopsy, the diagnosis of MASLD can be suspected on simple indices, such as the fatty liver index (FLI), combining body mass index (BMI), waist circumference, TG, and gamma-glutamyl transferase (GGT) level (33). Steatotic liver disease can also be suggested by elevation of transaminases, aspartate aminotransferase (AST) and especially alanine aminotransferase (ALT) (34). Besides liver enzymes (GGT, ALT) and TG, fasting insulin, SAU levels (35), or surrogate indices of IR including HDL-C (36) have also been shown to be independent predictors of MASLD. This is an additional reason to perform these blood tests (fasting insulin, HDL-C, and SAU).

Chronic liver disease with elevated transaminases and/or GGT may also reveal other conditions known to be associated with SFN, such as chronic alcohol abuse, hemochromatosis, sarcoidosis, or hepatitis C virus (HCV) infection.

Chronic alcohol abuse is a possible cause of SFN (37, 38) and in addition to elevated GGT, usually associated with AST/ALT ratio increase (> 2), an elevated MCV is also a validated biomarker for this diagnosis (39), justifying again the inclusion of CBC in the first-line workup for research on the possible etiology of SFN.

Hereditary hemochromatosis, mainly involving mutation of the HFE gene, is an abnormal accumulation of iron in the body, notably in the liver, often associated with diabetes, and which has been shown to be a possible cause of SFN (40), possibly underestimated. The diagnosis may be suspected on routine blood testing, by the elevation of transferrin saturation (TSAT), ferritin level, and MCV, while transaminases are elevated only in the case of secondary liver fibrosis (41).

Like hemochromatosis, sarcoidosis is a cause of asymptomatic liver disease with more frequently abnormal liver function tests (LFTs) (42). Indeed, LFT abnormalities can be observed in 20–40% of patients with sarcoidosis, mainly involving an increase in serum alkaline phosphatase (ALP) and GGT (43, 44).

Regarding HCV infection, the AST/ALT ratio is increased (>2) at an early stage, but is rather low in later stages, while the fibrotic stage can be suspected on an elevated AST/platelet ratio (APRI) or fibrosis index-4 (FIB-4 = (age × AST)/(platelet × √[ALT]) (45–47).

Thus, to perform a complete biological screening of liver diseases possibly associated with SFN, the assessment must include LFTs (AST, ALT, GGT, ALP), but also TSAT, ferritin, and CBC (for MCV and platelet count).

### Thyroid disease

Hypothyroidism, diagnosed by elevated thyroid-stimulating hormone (TSH) levels, is a possible cause of SFN usually mentioned (48, 49), although the prevalence and pathophysiological mechanisms of neuropathic lesion remain to be established in this context.

## Immunological, inflammatory, and infectious causes of SFN

### Autoimmune connective tissue disorders

Autoimmune connective tissue disorders are probably the main identifiable possible cause of SFN along with metabolic disorders in terms of prevalence, with, for example, a prevalence of positive antinuclear antibodies (ANA) in 27.5% of patients reported in the series of Lang et al. (5) versus 25% of elevated FPG (prediabetes) and 24.7% of elevated TG (metabolic syndrome) (Table 1). The main disorders associated with SFN are primary Sjögren’s syndrome (pSS) (50, 51) and systemic lupus erythematosus (SLE) (52). The first-line screening tests for these autoimmune disorders include ANA and anti-extractable nuclear antigen antibodies (ENA) (anti-Ro/SSA, anti-La/SSB, anti-Sm, anti-RNP, anti-Jo1, anti-Scl-70 (anti-topoisomerase I), and anti-centromere (CENP-B) antibodies) (53, 54).

### Celiac disease

Celiac disease is an autoimmune disorder primarily affecting the small intestine associated with intolerance to gluten. This is a possible cause of SFN usually mentioned, perhaps related to a malabsorption syndrome, but its prevalence is difficult to determine and maybe underestimated (55, 56). Symptoms and laboratory abnormalities mainly include abdominal symptoms (diarrhea, constipation, pain, or bloating sensation) and iron deficiency anemia (57, 58). Therefore, serum ferritin level and CBC are useful for the diagnosis. Screening for celiac disease in the absence of characteristic symptoms or signs is usually not recommended (58). However, as for hemochromatosis and sarcoidosis, celiac disease may be poorly symptomatic and screening may be justified in the context of SFN even in the absence of clinical warning signs. Blood assay of anti-tissue transglutaminase (anti-tTG) IgA is the first-line test for the diagnosis of celiac disease in patients without IgA deficiency with a sensitivity of 89–93% and a specificity of 98% (57–59).

### Monoclonal gammopathy

While amyloid light-chain (AL) amyloidosis is a definite cause of SFN, it is often difficult to establish a specific causal relationship between a monoclonal gammopathy (either of undetermined significance (MGUS) or associated with smoldering or multiple myeloma) and SFN in the absence of evidence of amyloidosis, even though numerous clinical cases have been reported (60–63). There is also controversy in the algorithmic approach to the diagnosis of monoclonal gammopathies. It is proposed, on the one hand, a stepwise approach, starting with serum protein electrophoresis (SPEP) as a first-line screening test, then possibly complemented by serum protein immunofixation electrophoresis (SIFE), serum free light chain assay (SFLCA), and urine tests based on medical record data (64); or, on the other hand, the systematic combination of SPEP, SIFE and SFLCA, as recommended by the International Myeloma Working Group (65, 66). This second approach is more likely justified as a first-line workup in the setting of SFN, since monoclonal peaks may be too weak in this condition to be discernible on SPEP and the main problem is rather related to AL amyloidosis, requiring the inclusion of SFLCA (67).

### Sarcoidosis

Sarcoidosis has been established as main cause of SFN (68, 69), but probably more because small fiber tests have been performed in patients with sarcoidosis, than because the diagnosis of sarcoidosis has been searched in patients with SFN. However, sarcoidosis is often asymptomatic or minimally symptomatic and this diagnosis justifies being systematically considered in the first line, as a possible cause of SFN. The best diagnostic approach is probably based on chest radiology, because pulmonary forms of sarcoidosis are clearly predominant (70). No biological marker is sensitive or specific to confirm the diagnosis of sarcoidosis. Serum angiotensin converting enzyme (ACE) level, in particular, appears to be particularly insensitive and nonspecific, and its elevation has been shown to be too frequent and clinically meaningless in a large series of patients with SFN (5). However, ACE values > 2 times the upper limit of normal should be considered relevant (70). Other biological criteria suggestive of sarcoidosis are hypercalcemia, polyclonal hypergammaglobulinemia on SPEP, or lymphopenia on CBC (70).

### Viral infections

The main viral infections causing SFN are probably due to HCV (71, 72) (but not hepatitis B virus) and human immunodeficiency virus (HIV) (73, 74) and the corresponding serologies should be included in the first-line diagnostic workup.

## Second-line blood tests

Other blood tests correspond to well-defined possible etiologies of SFN, but which complement first-line screening tests or are non-routine tests, prescribed in hospital setting by specialists and performed in specific laboratories, or are associated with specific clinical warning signs.

## Metabolic causes of SFN

### Diabetes and prediabetes

As mentioned above, the diagnosis of diabetes or prediabetes is based on FPG, HbA1C, and 2hPG values. While FPG and HbA1C can be considered as first-line tests, the indication and method of OGTT is more questionable. In fact, OGTT is useless if the diagnosis of diabetes or prediabetes is established on FPG and/or HbA1C values. Thus, OGTT is often only performed in case of borderline FPG or HbA1c values, with only a 2hPG measurement, except in the case of screening for gestational diabetes. In the context of SFN, it seems legitimate to us to consider this test as a second option even in the case of normal FPG and HbA1c values., as recommended in a variety of clinical conditions, including “unexplained neuropathy” in general (75). In addition, 1 h plasma glucose (1hPG) value during OGTT was shown to have superior value than conventional tests (FPG, A1C, and 2hPG) in predicting diabetes and associated complications (76). The OGTT must therefore include both 1hPG and 2hPG measurements.

### Vitamin B disorders

As mentioned above, there is a possible inaccuracy regarding the diagnosis of vitamin B12 deficiency based on the total circulating B12 level for values ≥180 pg./mL and ≤350 pg./mL (29). In this case, it will be necessary to measure holotranscobalamin (holoTC), which is unfortunately not available in all countries, methylmalonic acid (MMA), and total homocysteine (tHcy) in serum or plasma. An elevated plasma MMA level > 280 nmol/L is specific for B12 deficiency in patients younger than 65 years with normal renal function, while advanced age and impaired renal function may lead to an increase in MMA. The sensitivity of this test is also questionable in clinical practice. In contrast, an elevated plasma tHcy level > 11 μmol/L (with gender-dependent normative values) is a sensitive but nonspecific indicator of B12 deficiency, as tHcy is also influenced by vitamin B6 and folate (vitamin B9) status. Therefore, serum folate levels should also be measured during this second-line testing (29).

On the other hand, the search for the origin of vitamin B12 deficiency highlighted in the first- or second-line screening tests will be the subject of a specific assessment (for the diagnosis of malabsorption or intrinsic factor disorder), but which goes beyond the objective of this work.

### Alcohol abuse

Regarding alcohol abuse, beyond liver enzymes and MCV, carbohydrate-deficient-transferrin (CDT) and phosphatidylethanol (PEth) levels can be measured if chronic alcohol abuse is suspected and not admitted (39, 77).

## Immunological, inflammatory, and infectious causes of SFN

### Autoimmune connective tissue disorders

In the case of clinical features compatible with autoimmune connective tissue disorders and the absence of positivity of first-line tests (ANA and ENA), second-line screening tests include anti-double-stranded (anti-native) DNA antibodies (anti-dsDNA), C3, C4, and CH50 haemolytic units of the complement and also detection of cryoglobulins (53, 54). In addition to pSS and SLE, other autoimmune connective tissue disorders are possibly associated with an increased prevalence of SFN, including rheumatoid arthritis (RA) (78, 79) and systemic sclerosis (SSc, scleroderma) (80, 81).

Therefore, in patients with joint pain and swelling, it is necessary to add the following tests: C-reactive protein (CRP), rheumatoid factor (RF) and anti-cyclic citrullinated peptide antibodies (anti-CCP) for the diagnosis of RA (82). In patients with Raynaud’s phenomenon, telangiectasia, puffy fingers, or skin thickening of the fingers, it is necessary to complete the search for the diagnosis of SSc (83, 84) with an immunoassay for other SSc-related autoantibodies (anti-CENP-A/B, anti–RNA polymerase III (RP 11 and 155), anti-fibrillarin, anti-Ku, anti-90-kd nucleolar protein (NOR-90), anti-Th/To, anti-PM/Scl-75, anti-PM/Scl-100, anti-Ro52/tripartite motif-containing protein 21 (TRIM21), and anti-platelet-derived growth factor receptor (PDGFR) antibodies) beyond ENA screening (anti-Scl-70 anti-CENP-B antibodies) (85). This assessment can be extended to include the search for anti-ribonucleoprotein U1 (U1-RNP) antibodies for the diagnosis of mixed connective tissue disease (MCTD), which shares clinical characteristics with SLE, RA, and SSc (86).

### Other autoimmune disorders

Among other autoimmune diseases possibly associated with SFN, vasculitic mechanisms must also be considered. The clinical picture is then more likely acute, painful, distal, and asymmetrical (mononeuropathy multiplex pattern). In these cases, antineutrophil cytoplasmic antibody (ANCA) or cryoglobulinemia testing should be performed, as these have been associated with specific involvement of small nerve fibers in some cases (87–90).

In recent years, new antibodies associated with sensory neuropathies, including SFN, particularly presenting as acute-onset forms, have been discovered. Although their pathogenicity remains to be established and only a few laboratories perform these tests, the following antibodies warrant inclusion in advanced research on the possible etiology of SFN: anti contactin-associated protein-like 2 (anti-Caspr2), anti-leucine-rich glioma-inactivated 1 (anti-LGI1), anti-fibroblast growth factor receptor 3 (anti-FGFR3), anti-trisulfated heparin disaccharide (anti-TS-HDS), and anti-plexin D1 (91–95). The circumstances leading to the search for these antibodies remain to be defined, but probably include painful and especially acute-onset forms of SFN (94).

### Sarcoidosis

The diagnosis of sarcoidosis remains a challenge in clinical practice due to the heterogeneity of signs and symptoms and the lack of sufficiently sensitive and specific biomarkers to establish a diagnosis on their own. In addition to the standard blood tests offered as a first-line approach, such as ACE testing, whose limitations in terms of sensitivity and specificity have already been mentioned, it is worth noting that new biomarkers have been proposed with possible higher diagnostic sensitivity (96, 97). These biomarkers include 1,25-dihydroxy vitamin D (1,25(OH)_2_D or calcitriol), chitotriosidase (CTO), and soluble form of the interleukin 2 receptor (sIL-2R). Elevated serum level of 1,25(OH)_2_D, the hormonally active form of vitamin D, is associated with disease activity because granulomas are a source of extrarenal calcitriol production, but it also has diagnostic utility, particularly in the context of extrapulmonary forms of sarcoidosis (98), associated with hypercalcemia and hypercalcuria (99). A high serum level of CTO is also correlated with disease activity and multi-organ dissemination (100) with a diagnostic sensitivity of up to 89% (96), and the same is true for sIL-2R (101). Besides sarcoidosis, an increase in serum sIL-2R levels can also be observed in various immunological diseases possibly associated with SFN, such as SLE, RA, or ANCA-associated vasculitis (102). These tests may be offered in uncertain cases of suspected sarcoidosis, either clinically or based on chest radiological findings, although histopathological examination with the identification of granulomatous lesions remains the reference method for a definitive diagnosis (103).

## Hereditary causes of SFN

Genetic testing will always be based on specific clinical pictures and never performed in first-line workup. The following mutations can be searched:

- transthyretin (*TTR*) gene mutation in the case of clinical symptoms (dysautonomia, cardiomyopathic involvement, rapidly evolving distal polyneuropathy) compatible with familial amyloidosis (104–106);
- globotriaosylsphingosine (lyso-Gb3 or -GL3) serum level for men or alpha-galactosidase A (*GLA*) gene mutation for women in the case of clinical symptoms (paroxysmal painful acroparesthesiae in familial or child-onset context, cardiomyopathic involvement, proteinuria, dyshidrosis, angiokeratoma) compatible with Fabry disease (107–109);
- Sodium voltage-gated channels Nav1.7–1.8-1.9 (*SCN9A*-*SCN10A*-*SCN11A*) and transient receptor potential ankyrin 1 (*TRPA1*) gene mutations in the case of clinical symptoms (paroxysmal painful acroparesthesiae or erythermalgia in familial or child-onset context) compatible with for gain-of-function axonal channelopathies (110);
- collagen type VI alpha 5 (*COL6A5*) gene mutation for familial neuropathic itch (111).
- mutations in at least 12 different genes in the case of prominent sensory loss and ulcerative mutilations in combination with variable autonomic disturbances in hereditary sensory and autonomic neuropathies (HSANs) (112, 113).

Dried blood spot testing can be used as initial screening method for genetic analysis of *TTR* mutation (114) or for lyso-Gb3 level or alpha-galactosidase A activity measurement for the diagnosis of Fabry disease (115, 116).

## Other laboratory tests

Besides blood tests, the most relevant laboratory test in the investigation of the possible etiology of SFN is probably minor salivary gland biopsy (MSGB), which is particularly useful for the diagnosis of pSS, sarcoidosis, and amyloidosis (117). Skin biopsy can also have significant diagnostic yield in detecting amyloid deposits (7). Abdominal fat tissue aspirate or biopsy can also be used for screening immunoglobulin light chain (AL) amyloidosis, with a probability of finding deposits ranging from 70 to 90%, higher than for TTR amyloidosis (probability of finding deposits being 67% in the hereditary variant form and only 14% in the wild-type form) (118). Digestive tract biopsies from oesophago-gastro-duodenoscopy (OGD) are useful for the diagnosis of malabsorption and celiac disease (57, 58) and from rectoscopy for the diagnosis of amyloidosis (118). Other biopsy options for the finding of amyloid deposits include organ biopsy (heart, kidney) and transverse carpal ligament biopsies during carpal tunnel syndrome surgery (118).

Regarding medical imaging, chest X-ray or computed tomography (CT) scan is abnormal in 85–90% of patients with sarcoidosis (70). Transthoracic echocardiography (TTE) is useful for the diagnosis of cardiac amyloidosis or Fabry’s disease (119). Other imaging studies may be performed for the diagnosis of hepatopathy or myelopathy, for example, which may be present in various possible causes of SFN.

Specific tests can be used for the diagnosis of pSS, such as Schirmer’s test, ocular staining, and sialography (53). Finally, urinary tests are useful for the detection of kidney disease or monoclonal gammopathy (64).

## Conclusion

Despite a comprehensive screening strategy for identifying possible causes of SFN, many SFN cases remain “idiopathic,” with no identified cause, probably in half of the patients. The discovery of new autoantibodies offers hope, but their pathogenicity remains to be demonstrated (91, 95). The key question concerns the appropriateness and timing of repeating etiological testing. This will be guided by the occurrence of new clinical signs, but repeating this assessment at 1 year after initial testing can be considered if SFN symptoms persist. When no cause is identified, the therapeutic management strategy remains based solely on symptoms.

In clinical practice, the interpretation of complementary tests must always be cautious before concluding that there is a causal link between the detected abnormalities and the presence of SFN. The presence of risk factors for SFN concomitant with a clinical presentation of SFN does not necessarily mean causality. It may indeed be a coincidental association, linked to the high prevalence of this factor in the general population, particularly in relation to age. This concerns various metabolic factors or the presence of a monoclonal gammopathy, for example, as the prevalence of MGUS not associated with neuropathy increases with age (120). Similarly, with age, the prevalence of comorbidities increases, and the finding of abnormal tests may be more incidental than causal. It is important to emphasize that the relevance of complementary test results can be called into question if they are not correctly interpreted by the clinician. The etiology of SFN should be defined on a body of concordant evidence, grounded in relevant correlations between an identified possible cause and clinical manifestations, as well as the patient’s history in each particular case.

**Figure 1:**
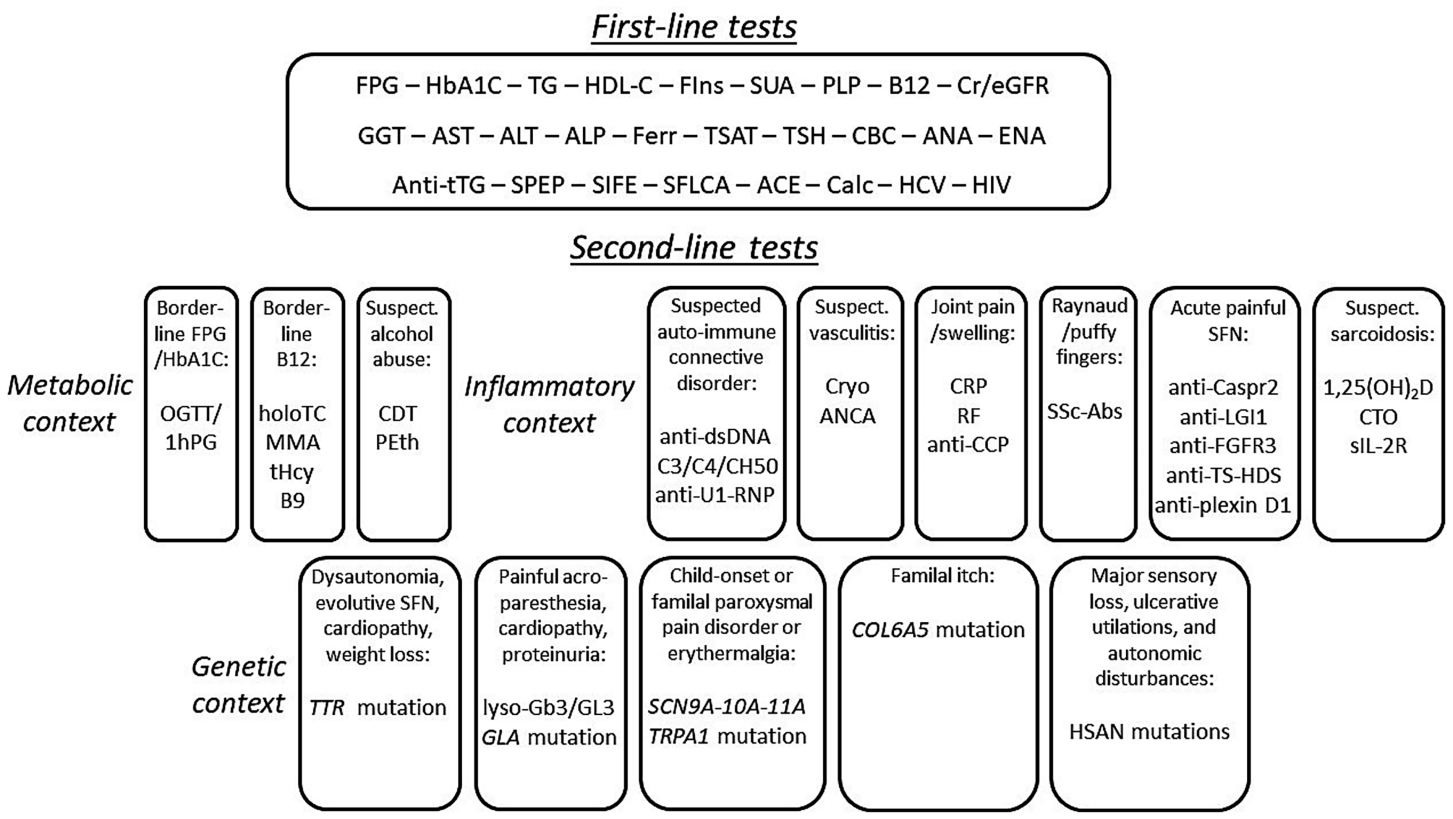
Algorithm flowchart for the sequential investigation of blood tests for screening the possible causes of small fiber neuropathy. The meaning of the acronyms can be found in Tables 2, 3.

This work is based neither on a systematic review nor a meta-analysis of the literature, but on the experience and recommendations of a small group of experts. This introduces biases and limitations, despite references to a body of literature addressing certain relevant aspects of the clinical contexts discussed. Consequently, the level or hierarchy of evidence supporting each test, as well as the definition of first- and second-line examinations, retains a degree of subjectivity (e.g., on the prevalence of the possible causes, the diagnostic yield of the tests, their cost, or their accessibility). However, we believe that this proposed list of tests (summarized in Figure 1) to explore the possible etiologies of SFN reflects a pragmatic view of reality and can prove very useful in clinical practice for the management of patients with SFN.
